# A phenomenological interview study with patients being non-conveyed in the ambulance service

**DOI:** 10.1186/s12873-023-00797-8

**Published:** 2023-03-16

**Authors:** Jakob Lederman, Caroline Löfvenmark, Therese Djärv, Veronica Lindström, Carina Elmqvist

**Affiliations:** 1 Department of Clinical Science and Education, Södersjukhuset, Karolinska Institutet, Stockholm, Sweden; 2 Academic Emergency Medical Services/AISAB Ambulance care in Greater Stockholm Ltd, Region Stockholm, Sweden; 3 Department of Health promoting science, Sophiahemmet University, Stockholm, Sweden; 4 Department of Medicine Solna, Karolinska Institutet, Stockholm, Sweden; 5 Department of Neurobiology, Care Sciences and Society, section of nursing, Karolinska Institutet, Stockholm, Sweden; 6 Samariten Ambulance, Stockholm, Sweden; 7 Department of Health and Caring Sciences, Linnaeus University, Växjö, Sweden; 8 Centre of Interprofessional Cooperation within Emergency care (CICE), Linnaeus University, Växjö, Sweden; 9 Department of Research and Development, Region Kronoberg, Växjö, Sweden

**Keywords:** Patients’ perspectives, Emergency medical services [MeSH], Ambulance care, Non-conveyance, Caring encounter, Phenomenology

## Abstract

**Background:**

Non-conveyed patients (i.e. patients who are not transported to a hospital after being assessed by ambulance clinicians) represent a significantly increasing proportion of all patients seeking ambulance care. Scientific knowledge about patients’ non-conveyance experiences is sparse. This study describes the lived experiences of non-conveyed patients in an ambulance care context.

**Methods:**

A reflective lifeworld research (RLR) approach founded on phenomenology is used. Data is derived from nine in-depth interviews with patients not conveyed by the ambulance service in a major Swedish region.

**Results:**

Patients’ lived experiences of becoming acutely ill or injured and not conveyed by ambulance to a hospital are characterised by several dynamic movements: losing and regaining situational and bodily control, dependence and autonomy, external competence and inner knowledge, handing over and regaining responsibility, and fear and security.

**Conclusions:**

Patients’ lived experiences of non-conveyance are complex and versatile. Although non-conveyed patients initially experience strong fear and the loss of situational and bodily control, they gradually feel more secure when experiencing confirmation and trust, which evolves into insecurity and uncertainty. The non-conveyance situation’s complexity from a patient’s perspective implies the need for ambulance organisations to take measures to prevent further suffering. Non-conveyed patients must be taken seriously in their unique situations, requiring ambulance clinicians to reflect and act with a conscious ethical perspective before, during and after their visit.

## Background

The ambulance service is a component of the prehospital emergency chain. Non-conveyed patients (i.e. those not transported to a hospital following assessment by an ambulance clinician [AC]) now represent a significantly increasing proportion of patients seeking ambulance care in the Western world [1–4]. Conducting non-conveyance assessments is considered more challenging and complex than conveying patients to an emergency department (ED) [5–7]. Previous non-conveyance research has focused on measuring patient outcomes, describing the non-conveyance care process and the non-conveyance population [4, 8, 9]. However, scientific knowledge about how patients experience non-conveyance is limited [10]. This knowledge is fundamental for developing non-conveyance that focuses on patient safety.

Patients who become sick or injured and in need of ambulance care are often vulnerable, exposed and dependent upon ACs [11, 12]. However, studies do not include the perspectives of non-conveyed patients. The complexity surrounding non-conveyance can be partly understood through different professional expectations; the concept of non-conveyance deviates from ACs expectations of working in the ambulance service, which typically focuses on acute illness and trauma [5, 6]. Swedish ambulance organisations are nurse-based, although several Swedish regions have increased formal requirements by stipulating that at least one specialist nurse should work in every ambulance unit. Swedish specialist nurse training programmes have been shown to disregard training targeted at exclusively non-conveyance cases [13]. Conversely, ACs view the performance of non-conveyance assessments as a high-responsibility task [5] as it potentially involves risk-taking, which could potentially harm the patient if they are incorrectly assessed. Compared to patients conveyed to an ED, non-conveyed patients are more often exposed to risks, such as delayed care, poor outcomes and even death if ACs fail to identify a serious underlying health problem [14–16]. In addition, performing non-conveyance assessments means acting in a clinical reality complicated by three paradoxes: the responsibility paradox, where the increased responsibility that comes with conducting non-conveyance assessments is not met with sufficient organisational support; the educational paradox, where ACs lack necessary and recurrent education focused on non-conveyance; and the feedback paradox, where the need to learn from previously conducted non-conveyance assessments is not sufficiently met, potentially negatively influencing future non-conveyance assessments [6].

Patient safety in a non-conveyance context has previously been evaluated by measuring outcomes through different endpoints following non-conveyance assessments, such as subsequent ED visits, hospital admissions and mortality [4, 9]. However, such measurements do not include patients’ perspectives on subsequent healthcare contacts and what these outcomes mean for the individual. Including patients’ perspectives on health care interventions offers a more meaningful understanding of the phenomenon [17].

Patients assessed by ambulance clinicians as non-urgent must be taken seriously. Otherwise, personal autonomy could be violated, causing suffering [18]. However, there remains a lack of in-depth scientific knowledge concerning patients’ lived experiences of non-conveyance [10].

Consequently, this study aims to describe non-conveyed patients’ lived experiences in an ambulance care context.

## Methods

### Study design

This study employs a phenomenological approach using a reflective lifeworld research (RLR) method [19]. To describe phenomena as they are lived and experienced by individuals, this research approach could clarify the essential meaning and variations of the discussed phenomenon, which in this study is “becoming acutely ill or injured and once assessed by ambulance clinicians, being non-conveyed and thus not accompanying the ambulance to a health care facility”. The researchers’ understanding of the phenomenon can be deepened through the methodological principles of the RLR approach: flexibility, openness and bridling [19, 20]. This study is described using the COREQ checklist for reporting qualitative research [21].

### Study setting and participants

The study’s target area had a population of approximately 2.2 million [22]. Annually, the ambulance service performs over 210,000 assignments, of which 14% result in non-conveyance [3]. It is a publicly funded health care service that patients can reach through the national emergency number. At least one of the two ambulance clinicians should be a registered nurse according to national regulations. Regional directives also require one of the two clinicians to have undergone one year’s additional university training and hold a specialist nursing degree [23]. The specialist nurse is held medically responsible within the ambulance team [24]. Furthermore, a patient-consent requirement is stipulated within the non-conveyance guidelines used by the ambulance service in the target study area [25]. The inclusion criteria for the study participants were non-conveyed patients who could speak either Swedish or English. A selection template was constructed to cover variations in phenomena judged to be significant: the patient’s geographical location (highly urban, urban or rural), gender, age and chief complaint, the ambulance company and whether the assignment was carried out during the day or night and on a working day or at the weekend. The exclusion criteria were individuals aged < 18 years, those clearly influenced by alcohol and or narcotics and patients who could not fully understand oral and or written information about the study (e.g. due to cognitive impairment). The inclusion and exclusion criteria were applied by ambulance clinicians conducting the non-conveyance assessment. The variations covered in the selection template were all met.

The heads of department in all three ambulance companies approved the study’s implementation before data collection was initiated. Written and verbal information about the study was distributed to all three companies. The ambulance clinicians were required to present the study to non-conveyed patients meeting the inclusion criteria once the non-conveyance decision had been made. The clinicians also asked the patients if they were interested in receiving further information about the study via telephone in the subsequent days. Eleven potential participants were asked to take part in the study, two of whom declined due to ongoing illnesses. Another patient did not offer a reason for refusing to participate. Five of the participants were female. The participants’ median age was 67 years (range 35–95 years) (Table 1).

**Table 1:** Participants’ characteristics

No	Sex	Age	Medical complaint	Geographical location	Assignment time of day	Ambulance company
1	Male	59	Unconsciousness	Rural	Evening	1
2	Male	82	Choking	Rural	Day	1
3	Male	67	Chest pain	Urban	Day	2
4	Female	35	Chest pain/breathing difficulties	Highly urban	Night	2
5	Female	77	Chest pain/breathing difficulties	Urban	Day	3
6	Male	86	Hypoglycaemia	Urban	Evening	3
7	Female	95	Headache/amnesia	Highly urban	Day	2
8	Female	64	Dizziness/nausea	Urban	Evening	2
9	Female	61	Visual impairment	Urban	Night	2

### Data collection

The methodological principles of flexibility, openness and bridling were actively implemented throughout the research. The researchers’ awareness of their preconceptions about the phenomenon in question was developed throughout the study [19]. Bridling was made possible through self-awareness, trying not to understand participants’ experiences too quickly and questioning the first author’s preconceptions about the phenomenon. Data were collected via in-depth, open-ended individual interviews conducted between 01/02/2019 and 15/02/2020. The first author conducted and digitally recorded the interviews in Swedish. The interviews were carried out at locations chosen by the participants. All were native Swedish speakers. The interviews began with the same open question: “Please tell me about the situation when you became acutely ill and once assessed by ambulance clinicians, did not accompany the ambulance to a health care facility”. Openness and flexibility towards the participants’ experiences of the phenomenon characterised the interviews. Follow-up questions concerning the phenomenon, such as “Please expand on this perspective” or “You mentioned fear, please tell me more”, were used repeatedly during the interviews. The interviews varied between 14 and 56 min in length, with a median of 34 min. Subsequently, the first author performed verbatim transcription.

### Data analysis

Data analysis was performed in Swedish primarily by the first author, with support from two co-authors. It was conducted following the RLR approach, using the aforementioned methodological principles [19, 20]. The analysis was characterised by a recurrent movement between the initial whole, the constituent parts and the new whole, to categorise concrete lived experiences into abstract levels and thereby explicate the phenomenon’s essence. The interview transcripts were read several times and subsequently divided into smaller parts, called “meaning units”, related to the phenomenon of interest. Each unit’s meaning was described, and groups of meanings that were related based on similarities and differences were abstracted into “patterns”. The patterns were repeatedly compared using a process called “figure-background-figure”, which facilitates the discovery of new insights and perspectives (Dahlberg, 2008), and then abstracted into “clusters”. This process involved asking questions partly targeted at the data material, such as “What does this cluster mean in comparison to this cluster” and partially as “How come I see this meaning in this way?” As the construction of clusters and the abstraction process diverge from the original text, the dynamic processes of the “parts and the whole” aim to ensure that the data material is not distorted. Furthermore, a description of the studied phenomenon’s essence is provided as one cluster is viewed in relation to another [19]. The studied phenomenon’s essential meaning as experienced by the participants is the essence: a meaning that does not vary. The phenomenon’s essence and its variances, the constituents, including the quotations, are presented in the following sections.

## Results

The essential meaning underlying becoming acutely ill or injured and not being conveyed by ambulance to a hospital is characterised by several dynamic movements of physical, psychological, and emotional phases: losing and regaining situational and bodily control, fear and security, dependence and autonomy, external competence and inner knowledge, and handing over and regaining responsibility. A loss of situational and bodily control evolves from sudden physical deterioration. Dependence on others ensues from vulnerability with existential fear, an inability to act and a decreased level of autonomy. An ongoing dynamic movement between fear and security progresses before, during and after the non-conveyance experience. Responsibility is initially transferred once help arrives at the scene. A need for support in a chaotic situation partly characterises the experience.

Developing trust requires faith in the helpers’ external competence and professional assessment. Physical assessment reduces fear, and feelings of security evolve. Moreover, respectful dialogue enhances the process of regaining control over the situation. Clinicians who show interest in the patient allow feelings of being seen and heard to develop; hence being regarded as capable enhances the process of regaining bodily control. Reclaiming autonomy and independence requires support. Once the non-conveyance decision is taken, the responsibility is handed back by the clinician and received by the patient at the end of the experience. However, once the patient is alone again, the responsibility now rests upon their inner knowledge and perceived ability. An oscillation between security and uncertainty is revealed by the end of the non-conveyance experience.

### Being acutely ill or injured

Becoming acutely ill or injured and not being conveyed by ambulance to a hospital often begins with a sudden physical deterioration in which the patient experiences an impending existential threat. The strength of this fear causes the patient to experience a sudden pause in life, as if time has stopped. Their ability to act is impaired to the extent that they become dependent on others to decide whether to call an ambulance. The patient perceives their bodily functioning to be limited due to the ongoing physical symptoms, which catalyses existential thoughts and fear:

I felt … now I’m dying … but I continued out and tried to go as far as possible and when I got out, I sat down on the couch there and took a pillow and held it very hard against my chest. (Participant 5)

Dependence on others is further described as seeking someone else’s opinion regarding the symptoms’ severity when the patient does not recognise their body. Not having to decide whether an ambulance should be requested was perceived by the patients to be reassuring; someone else had assessed the situation and made the decision. New or more severe symptoms complicated the situation. Sudden physical deterioration also reminded patients of previous experiences of living with their severe diseases or those of a significant other, increasing the perceived fear:

You could say that I suddenly felt weird in my head if I say so. And then I thought about … that my husband got a stroke. And I thought, ‘Oh my god, it must not happen to me”. (Participant 7)

Perceived fear is often shared with significant others or individuals close to the patient. Hence, significant others carry a heavy responsibility before the ambulance clinicians arrive. Some patients call the non-emergency medical helpline in search of guidance. Fear consumes some patients when the word “ambulance” is mentioned, as needing an ambulance implies severe illness or injury. Moreover, the patient’s view of the situation does not always correspond with that of the healthcare call centres:

I became a bit shocked actually because then I suddenly thought, “Is it that dangerous?“ That was it. So … I mean … you think that you can be in pain and things can happen, but it cannot go too far either because then it will be scary, do you understand? So … yes … no … I had not actually thought of an ambulance. So yes … I became a bit upset. (Participant 9)

### Being vulnerable

When patients become acutely ill or injured and are not conveyed by ambulance to a hospital, they slowly develop a sense of safety in a chaotic situation, even before the ambulance clinicians arrive at the scene. Knowing that the ambulance is on its way is reassuring in itself when the patient experiences substantial fear. Vulnerability is further experienced through the actions performed by ambulance clinicians. Even actions performed before meeting ambulance clinicians influence vulnerable patients. Hearing their footsteps gives the patient some security in a fearful and threatening situation. Once the ambulance clinicians arrive at the scene, the responsibility is handed over to them by either the patient or their significant other. From a patient’s perspective, the ambulance clinician demonstrates their acceptance of this responsibility by performing the actions related to their assessment. The situation taken seriously, alongside the clinicians’ actions, gives patients some security, initiating the process of regaining their inner strength and self-belief:

Yes, but that they actually investigate properly. It is not someone that just comes in and states that “there is nothing wrong with you”, but they actually check that you are okay. They do all the tests and all that ballet and hey, that’s great. It gave me security. Yes. That is good. (Participant 1)

In addition, patients can experience increased concern if they do not receive a specific physical examination, such as an ECG. In contrast, the fact that ambulance clinicians do not perform a specific examination can also be interpreted as positive based on the severity of the patient’s condition. Furthermore, time relates to patients’ expectations and view of ambulance care; it is linked to stress, time pressure and life-threatening conditions. Conversely, being given time during the non-conveyance encounter is also experienced positively:

And they had … what I thought was so good, was that they were so relaxed and nice, not in a hurry. Not in a hurry at all. They had time for me, which was very important for me in this situation, because you are quite upset when you feel like I felt: “Oh my god, now something is going on”. (Participant 7)

Previous negative experiences with ambulance clinicians can influence future encounters, including non-conveyance. Meeting ambulance clinicians with a genuine interest in helping increased the patient’s inner belief in their ability to manage the situation. Genuine interest was demonstrated by involving the patient in the dialogue:

She was interested in me as a person and not just getting the patient to the hospital and leave[ing] … Hm … they showed an interest in how I felt, and I did not get that feeling of load and go. As I felt the time before... when the ambulance conveyed me to the ED. (Participant 3)

### Active involvement in decision-making

When patients become acutely ill or injured and are not conveyed by ambulance to a hospital, they must be shown interest and involved in the dialogue; patients need to be addressed respectfully according to their unique situation. This dialogue is fundamental for patients’ decision-making, which is influenced by several external factors. A self-strengthening process is initiated through respectful dialogue. It facilitates the non-conveyance decision-making process within the patient and in dialogue with the ambulance clinicians. Patients consider an invitation to actively participate in the dialogue important when trying to regain control. Meeting ambulance clinicians who act calmly and seriously is conducive to patients’ sense of security:

Yes, they are professionals. They know what to do; they are not nervous. They come in completely calm, and then they do what they are supposed to. (Participant 2)

The process leading to a non-conveyance decision, taken by the patient in consensus with the ambulance clinicians, is influenced by several external factors: notions of overcrowded EDs with long waiting times, a prolonged wait for an ambulance (indicating a congested emergency health care system), social factors, such as having young children at home, and ambulance clinicians pronouncing their view of the situation. Furthermore, the patient’s dependence on ambulance clinicians becomes evident through the decision-making process. The patient is offered conveyance by ambulance to an ED, and the ambulance clinicians simultaneously give their opinion of the situation:

They offered me to come with them if I wanted to, but their assessment was that I did not need it. Then I felt … then I listened to them. (Participant 4)

The ambulance clinicians’ medical competence reportedly influences the non-conveyance encounter and decision-making process in several ways. On the one hand, patients found comfort and trust in their expertise. Listening to the ambulance clinicians’ explanations made the non-conveyance decision possible by enabling the patient to recover enough of their strength and inner belief. On the other hand, patients’ dependence due to lower competence appeared to prevent some patients from questioning the ambulance clinicians’ views of the situation and the absence of specific physical examinations. The fact that someone with superior medical prowess took their time and actively listened, decreased the imbalance of power by indicating to the patient that they were an important part of the dialogue:

They listened to me and yes … how do I say it … and did not come up with their own things, but they listened to me and processed what I said. So, I do not know how to say it, but it was a very good meeting with them. It felt very good. And it calmed me down. (Participant 9)

### Being safe in the aftermath and missing answers

When a patient becomes acutely ill or injured and is not conveyed by ambulance to a hospital, the responsibility is handed back to them by the ambulance clinicians at the end of the encounter. The ability to regain responsibility requires a significant degree of safety. However, once the patient is alone again, their feelings of security in the ambulance clinicians’ presence are soon replaced by doubt and, at times, missing answers. During non-conveyance encounters, patients experience various emotions and receive a considerable amount of information. The latter can be difficult to process and remember once the ambulance clinicians have left. Sometimes, the ambulance clinicians urged the patient to contact the Emergency Medical Command Centre (EMCC) or the ED in the event of deterioration, and in some cases, responsibility was handed over to a significant other. Nonetheless, it was not always clear to the patient or significant other what symptoms they should be aware of:

Then they told him to “make sure she calls back if she feels worse” or something. Kind of keep a little track of her, kind of. I did not really hear. … Yes, he was very tired the next day because he had not been able to sleep properly. … He was afraid it would happen again, that I would have pain again. That it would be something with the heart or something. (Participant 4)

The comfort and trust experienced in the presence of the ambulance clinicians can be replaced by feelings of uncertainty once they have left, as the responsibility now rests on the patient’s knowledge and ability, giving rise to sudden feelings of doubt. Several patients admitted to regretting the non-conveyance decision, as they were left not knowing what they had suffered from:

But it’s probably … that I probably should have accompanied in any case. To find out why my symptoms released. And especially when it felt like it was something that was dissolved when the pain released. Because I do not know if a clot in the pulmonary artery can dissolve itself and go unnoticed, so to speak. After all, it was something that gave me the breathing problems from the beginning. (Participant 3)

The ambulance clinicians referred several patients to their general practitioner following the non-conveyance encounter. However, some patients were met with questions and scepticism about their need for further care upon visiting their general practitioner. The support they felt during their encounter with the ambulance clinicians quickly changed to feelings of rejection and powerlessness. Conversely, a few patients were admitted to hospitals following the non-conveyance encounter. Despite the reasons for admissions being associated with the chief complaints during the non-conveyance encounters, the patients’ views on non-conveyance were nevertheless positive. Being given the option to avoid visiting the ED was highlighted as an important reason for this positive outlook, even in cases where the patient visited the ED the following day:

And so I was admitted for ten days. It was great; they decreased my general anxiety, and my blood sugar levels were normalised. After that, I came home, and it has gone well overall. (Participant 6)

## Discussion

### Interpretation of the results

The patients’ lived experiences of becoming acutely ill or injured and not conveyed by ambulance to a hospital have been revealed as a complex caring encounter consisting of several dynamic movements influencing the patients before, during and after the non-conveyance situation.

Research on patients’ perspectives in an ambulance service context has been performed to a lesser extent than research on ambulance clinicians’ experiences [26]. This notion also applies to research on non-conveyance. Our results are therefore of importance when trying to understand the increasing number of non-conveyed patients.

Individuals experience their lifeworld through the living body [27]. When acutely ill patients are not conveyed to a hospital following professional assessment by ACs, they initially experience vulnerability and existential fear. The patient’s lifeworld suddenly changes and they lose bodily control. From a phenomenological perspective, individuals cannot be separated from their bodies. This notion could explain the strong existential fear experienced by non-conveyed patients once they no longer recognise their bodies and thus experience a loss of situational and bodily control. These results support previous empirical findings [28] and hence constitute important aspects to consider when the goal is to create a caring encounter based on safety and trust.

Before and during the non-conveyance situation, patients become dependent on other individuals [28]. Several measures are taken before the EMCC is called, and patients seldom place the call as seeking help in the healthcare system means admitting to being vulnerable, powerless and weak [29]. Hence, our findings indicate that an ambulance is not called at the onset of physical deterioration but following a protracted process within the patient and dialogue with significant others. Moreover, non-conveyed patients seek confirmation from ambulance clinicians regarding their actions thus far (i.e. whether the patient or significant other has made an error when calling the EMCC). In the event of no such confirmation, non-conveyed patients have been found to experience a violation of their dignity and integrity [30]. The non-conveyed patients in our study experienced vulnerability and suffering when their condition was not taken seriously. This outcome reflects the significance of being respected by ambulance clinicians despite patients’ ailing conditions; these findings are congruent with previous research (Rantala et al., 2016; van Doorn et al., 2021). Our results further indicate the need for ambulance clinicians to be aware that events occurring before their arrival affect the non-conveyed patient. The fact that the majority of all non-conveyance assignments are classified as the highest priority by the EMCC [3] could reinforce the patients’ need to be taken seriously by ambulance clinicians once they arrive at the scene. Furthermore, suffering can increase if one part of the prehospital emergency chain performs an action that the patient interprets as serious and the second part does not. Similar courses of events could explain our finding that non-conveyed patients felt disregarded when they visited their general practitioner at the request of an ambulance clinician. These results support previous empirical findings demonstrating organisational shortcomings, often placing the prehospital emergency service on the periphery of the wider healthcare system (Knowles et al., 2018; Lederman et al., 2019). These findings call for further studies and measurements in daily clinical ambulance practice to create more favourable circumstances and dialogue between the ambulance service and the primary care system. Insecurity and powerlessness evolve when healthcare personnel dismiss a patient’s suffering in their unique situation [31]. In our study, this observation was corroborated by the non-conveyed patients who experienced increased situational and bodily control when shown interest through respectful dialogue, indicating that being taken seriously builds mutual trust in non-conveyed patients and ambulance clinicians. Trust is closely linked to vulnerability: the more vulnerable a patient feels, the greater the ambulance clinician’s responsibility to act attentively and establish trust [18]. In a study investigating deviation reports in an ambulance care context, non-conveyed patients revealed nonchalance and disinterest as two recurrent themes in descriptions of ambulance clinicians’ actions [30]. An absence of ethical dialogue resulting in disinterest in catering to the patient’s unique disposition is an individual and an organisational challenge that must be managed to create favourable circumstances for establishing a caring experience [30].

The act of handing over responsibility once ambulance clinicians arrive at the scene has been described previously [32, 33], although not in a non-conveyance context. Handing responsibility back to the patient at the end of the encounter distinguishes non-conveyance from other situations. Before handing back responsibility to a non-conveyed patient, ambulance clinicians assess the patient’s ability to manage this responsibility [6]. Furthermore, patients perceived receiving this responsibility as empowering. However, once they were alone again, several patients described the responsibility as burdensome, implying that ambulance clinicians must have the ability and organisational support to create safe environments for non-conveyed patients once they leave.

### Methodological considerations

An RLR study’s validity, objectivity and transferability are based on the methodological principles of the RLR approach [19, 20]. These principles were applied throughout this research. The first author’s preconceptions were bridled through self-awareness, attempting not to understand participants’ experiences too quickly and questioning the first author’s understanding throughout the research process. The interviews were conducted using the methodological principles and adhered to the studied phenomenon. Moreover, the research group organised recurrent seminars in which they reflected on the interviews, analysed the evolving results and discussed the conclusions drawn from them. An RLR study’s validity is influenced and strengthened by the high abstraction level of the phenomenon’s essence, that is, the description of the phenomenon is on an essential level [20].

A possible limitation influencing the study´s transferability is the participant recruitment process, which was largely based on the ambulance clinicians’ willingness to inform prospective participants about the study. We experienced recruitment difficulties throughout the inclusion process; ambulance clinicians frequently forgot to ask eligible non-conveyed patients if they would be willing to participate in the study. However, the procedures described for recruiting prospective participants reflect the only way to obtain contact information about non-conveyed patients. Directly contacting patients following the non-conveyance encounter could reduce selection bias and recruitment difficulties, excluding patient selection by clinicians. Although the variations covered in the selection template were met, transferability could be limited as patients seek ambulance care for diverse medical complaints. Generally, our data were deemed rich in content and variety. The material’s richness, covering several aspects of patients’ experiences of non-conveyance, both positive and negative, suggests that selection bias did not influence participant recruitment to the extent of significantly affecting the study’s transferability and validity. The participants were able to describe their lived experiences of the phenomenon of interest without difficulty relatively early in the interviews. A possible explanation for this finding is that the studied phenomenon is new and well-defined. Reflective lifeworld research with a high essential level of results is transferable to other care contexts [20], such as primary health care and ambulatory care [34].

## Conclusion

Patients’ lived experiences of non-conveyance are complex and versatile. After initially reporting a strong fear and loss of situational and bodily control, patients gradually felt more secure by experiencing confirmation and trust. However, once the patients were alone again, these feelings evolved into growing insecurity and uncertainty due to not knowing what they had suffered from. Non-conveyed patients must be taken seriously in their unique situations, requiring ambulance clinicians to reflect and act with a conscious ethical perspective before, during and after their visit. The complexity surrounding the non-conveyance situation from a patient’s perspective implies the need for ambulance organisations to take measures to prevent further suffering in non-conveyed patients. This finding justifies the need for further studies investigating how to manage the complexity of non-conveyance from the perspectives of patients, ambulance clinicians and ambulance organisations. Furthermore, the uncertainty that sometimes develops following a non-conveyance experience requires ACs to possess clinical abilities and organisational support to create safe environments for their patients.

## Declarations

### Data sharing statement

The datasets generated and/or analysed during the current study are not publicly available due the risk of undermining the participants’ anonymity but are available from the corresponding author on reasonable request.

## Abbreviations

AC: Ambulance clinician
COREQ: Consolidated criteria for reporting qualitative research
ED: Emergency department
EMCC: Emergency medical communication centre
RLR: Reflective lifeworld research

## References

[1] Magnusson C, Herlitz J, Axelsson C (2020). Patient characteristics, triage utilisation, level of care, and outcomes in an unselected adult patient population seen by the emergency medical services: a prospective observational study. BMC Emerg Med.

[2] Paulin J, Kurola J, Salanterä S, Moen H, Guragain N, Koivisto M (2020). Changing role of EMS -analyses of non-conveyed and conveyed patients in Finland. Scand J Trauma Resusc Emerg Med.

[3] Lederman J, Lindström V, Elmqvist C, Löfvenmark C, Djärv T (2020). Non-conveyance in the ambulance service: a population-based cohort study in Stockholm, Sweden. BMJ Open.

[4] Ebben RHA, Vloet LCM, Speijers RF, Tönjes NW, Loef J, Pelgrim T (2017). A patient-safety and professional perspective on non-conveyance in ambulance care: a systematic review. Scand J Trauma Resusc Emerg Med.

[5] Höglund E, Schröder A, Möller M, Andersson-Hagiwara M, Ohlsson-Nevo E (2018). The ambulance nurse experiences of non-conveying patients. J Clin Nurs.

[6] Lederman J, Löfvenmark C, Djärv T, Lindström V, Elmqvist C (2019). Assessing non-conveyed patients in the ambulance service: a phenomenological interview study with swedish ambulance clinicians. BMJ Open.

[7] Knowles E, Bishop-Edwards L, O’Cathain A (2018). Exploring variation in how ambulance services address non-conveyance: a qualitative interview study. BMJ Open.

[8] Mikolaizak AS, Simpson PM, Tiedemann A, Lord SR, Close JC (2013). Systematic review of non-transportation rates and outcomes for older people who have fallen after ambulance service call-out. Australas J Ageing.

[9] Yeung T, Shannon B, Perillo S, Nehme Z, Jennings P, Olaussen A (2019). Review article: outcomes of patients who are not transported following ambulance attendance: a systematic review and meta-analysis. EMA - Emergency Medicine Australasia.

[10] King R, Oprescu F, Lord B, Flanagan B (2020). Patient experience of non-conveyance following emergency ambulance service response: a scoping review of the literature. Australas Emerg Care.

[11] Holmberg M, Forslund K, Wahlberg AC, Fagerberg I (2014). To surrender in dependence of another: the relationship with the ambulance clinicians as experienced by patients. Scand J Caring Sci.

[12] Aléx J, Lundgren P, Henriksson O, Saveman BI (2013). Being cold when injured in a cold environment - patients’ experiences. Int Emerg Nurs.

[13] Sjölin H, Lindström V, Hult H, Ringsted C, Kurland L (2015). What an ambulance nurse needs to know: a content analysis of curricula in the specialist nursing programme in prehospital emergency care. Int Emerg Nurs.

[14] Lederman J, Lindström V, Elmqvist C, Löfvenmark C, Ljunggren G, Djärv T (2021). Non-conveyance of older adult patients and association with subsequent clinical and adverse events after initial assessment by ambulance clinicians: a cohort analysis. BMC Emerg Med.

[15] Coster J, O’Cathain A, Jacques R, Crum A, Siriwardena AN, Turner J (2019). Outcomes for patients who contact the emergency Ambulance Service and are not transported to the Emergency Department: A Data linkage study. Prehospital Emerg Care.

[16] Tohira H, Fatovich D, Williams TA, Bremner AP, Arendts G, Rogers IR (2016). Is it appropriate for patients to be discharged at the scene by paramedics?. Prehospital Emerg Care.

[17] Larson E, Sharma J, Bohren MA, Tunçalp Ö (2019). When the patient is the expert: measuring patient experience and satisfaction with care. Bull World Health Organ.

[18] Rantala A, Ekwall AR, Forsberg AR (2016). The meaning of being triaged to non-emergency ambulance care as experienced by patients. Int Emerg Nurs.

[19] Dahlberg K, Dahlberg H, Nyström M. Reflective lifeworld research. 2nd edition. Lund: Studentlitteratur; 2008.

[20] van Wijngaarden E, van der Meide H, Dahlberg K (2017). Researching Health Care as a meaningful practice: toward a nondualistic view on evidence for qualitative research. Qual Health Res.

[21] Tong A, Sainsbury P, Craig J (2007). Consolidated criteria for reporting qualitative research (COREQ): a. Int J Qual Health Care.

[22] Stockholm County Council. Patienttransporter i framtidens hälso-och sjukvård. 2016.

[23] Stockholm County Council. Årsrapport 2018 Prehospitala verksamheter i Stockholms läns landsting [Anual report 2018 Prehospital units in the Stockholm County Council]. 2018.

[24] Medical guidelines for the Ambulance service. Stockholm:Stockholm County Council; 2017.

[25] Medical guidelines for the (2017). Ambulance service - non conveyance.

[26] Ahl C, Nyström M (2012). To handle the unexpected - the meaning of caring in pre-hospital emergency care. Int Emerg Nurs.

[27] Merleau-Ponty M (2011). Phenomenology of perception.

[28] van Doorn SCM, Verhalle RC, Ebben RHA, Frost DM, Vloet LCM, de Brouwer CPM (2021). The experience of non-conveyance following emergency medical service triage from the perspective of patients and their relatives: a qualitative study. Int Emerg Nurs.

[29] Berglund M, Westin L, Svanström R, Sundler AJ. Suffering caused by care-Patients’ experiences from hospital settings.Int J Qual Stud Health Well-being. 2012;7. 10.3402/qhw.v7i0.18688 PMC3430007 22943888

[30] Ahlenius M, Lindström V, Vicente V. Patients experience of being badly treated in the ambulance service: A qualitative study of deviation reports in Sweden. 2017. 10.1016/j.ienj.2016.07.004. 10.1016/j.ienj.2016.07.004 27567212

[31] Dahlberg K (2002). Suffering from care – the unnecessary suffering. Nord J Nurs Res.

[32] Jepsen K, Rooth K, Lindström V (2019). Parents’ experiences of the caring encounter in the ambulance service—A qualitative study. J Clin Nurs.

[33] Elmqvist C, Fridlund B, Ekebergh M (2008). More than medical treatment: the patient’s first encounter with prehospital emergency care. Int Emerg Nurs.

[34] Coordinated development for good quality - local health care. God och nära vård [Good quality, local health care – a primary care reform]. Stockholm; 2018.

[35] World Medical Association. WMA Declaration of Helsinki - Ethical principles for medical research involving human subjects. 2013; June 1964:29–32.

